# Comparison of Accuracies between Real-Time Nonrigid and Rigid Registration in the MRI–US Fusion Biopsy of the Prostate

**DOI:** 10.3390/diagnostics11081481

**Published:** 2021-08-16

**Authors:** Sung Il Hwang, Hyungwoo Ahn, Hak Jong Lee, Sung Kyu Hong, Seok-Soo Byun, Sangchul Lee, Gheeyoung Choe, Jun-Sung Park, Yuri Son

**Affiliations:** 1 Department of Radiology, Seoul National University Bundang Hospital, Seongnam 13620, Korea; hwangsi49@gmail.com; 2 Seoul National University Bundang Hospital Program in Nano Science and Technology, Department of Radiology, Seoul National University College of Medicine, Seongnam 13620, Korea; hakjlee@gmail.com; 3 Department of Transdisciplinary Studies, Seoul National University Graduate School of Convergence Science and Technology, Suwon 16229, Korea; 4 Department of Urology, Seoul National University Bundang Hospital, Seongnam 13620, Korea; skhong@snubh.org (S.K.H.); ssbyun@snubh.org (S.-S.B.); slee@snubh.org (S.L.); 5 Department of Pathology, Seoul National University Bundang Hospital, Seongnam 13620, Korea; gychoe@snubh.org; 6 Samsung Medison, Seoul 05340, Korea; njs.park@samsungmedison.com; 7 Samsung Electronics, Hwaseong 18450, Korea; yuri.son@samsung.com

**Keywords:** fusion biopsy, nonrigid registration, rigid registration, elastic deformation, ultrasonography, magnetic resonance imaging

## Abstract

Magnetic resonance imaging (MRI) is increasingly important in the detection and localization of prostate cancer. Regarding suspicious lesions on MRI, a targeted biopsy using MRI fused with ultrasound (US) is widely used. To achieve a successful targeted biopsy, a precise registration between MRI and US is essential. The purpose of our study was to show any decrease in errors using a real-time nonrigid registration technique for prostate biopsy. Nineteen patients with suspected prostate cancer were prospectively enrolled in this study. Registration accuracy was calculated by the measuring distance of corresponding points by rigid and nonrigid registration between MRI and US, and compared for rigid and nonrigid registration methods. Overall cancer detection rates were also evaluated by patient and by core. Prostate volume was measured automatically from MRI and manually from US, and compared to each other. Mean distances between the corresponding points in MRI and US were 5.32 ± 2.61 mm for rigid registration and 2.11 ± 1.37 mm for nonrigid registration (*p* < 0.05). Cancer was diagnosed in 11 of 19 patients (57.9%), and in 67 of 266 biopsy cores (25.2%). There was no significant difference in prostate-volume measurement between the automatic and manual methods (*p* = 0.89). In conclusion, nonrigid registration reduces targeting errors.

## 1. Introduction

Prostate cancer (PCa) is by far the most common male cancer in the United States and second leading cause of death [1]. Transrectal ultrasound (US) is still regarded as the first imaging modality for the evaluation of the prostate because it is easy, fast to perform, and gives good information about prostate volume. However, US shows low sensitivity and specificity for the detection of cancer [2], which necessitates the systematic biopsy (SB) of the prostate rather than the targeted biopsy (TB) of a suspicious area. Multiparametric magnetic resonance imaging (MRI) has increasingly been used as the main imaging modality for prostate evaluation since the introduction of Prostate Imaging-Reporting and Data System (PI-RADS). In addition to its excellent staging ability, MRI is superior to other imaging modalities for the detection and localization of the PCa. Therefore, recently updated European Association of Urology guidelines strongly recommend multiparametric prostate MRI prior to biopsy regardless of the patient’s biopsy history [3].

The greatest advantage of MRI-based TB is a reduction in the false-negative rates for PCa detection, as suspected lesions are first identified in MRI before biopsy [4]. There are two ways for a TB of a suspicious lesion, the direct in-bore MRI-guided biopsy, and a fusion biopsy using US and MRI. The former has the advantage of not requiring registration, but requires two separate sessions of MRIs, and systemic biopsy cannot be applied before or after the TB [5]. Fusion biopsy is easy to perform with local anesthesia, takes less time, but the registration between two different imaging modalities is essential [6].

To achieve an accurate registration, the real-time correction of US with MRI is needed. The prostate gland is inevitably deformed by the transrectal probe during US examination, which is different from MRI using surface coil. Patient position and movement during the procedure can be another source of errors. Higher precision was reported with the deformable MRI-transrectal US registration system [7]. However, many US vendors currently either provide only rigid registration or a sophisticated elastic fusion system with retrospective nonrigid registration [8,9]. We developed a solution offering both rigid and real-time corrected nonrigid registration during the live biopsy without requiring an intermediate 3D US [10]. With this system, we show error reduction through real-time nonrigid registration technique for prostate fusion biopsies.

## 2. Materials and Methods

### 2.1. Patients

This single-centered, uncontrolled prospective study for patients with suspected PCa was approved by our institutional review board. Eligible patients were asked to voluntarily participate in this study after receiving oral and written information from a radiologist performing the examination.

The inclusion criteria were as follows:

- patients over 30 years of age;
- patients requested for the prostate fusion biopsy;
- patients with a PI-RADS score of 3 or higher;
- patients with signed informed consent.

The exclusion criteria were as follows:

- Contraindicated for prostate fusion biopsy
  ▪ patients with bleeding tendency;
  ▪ patients allergic to local analgesic agents;
  ▪ patients intolerant of prostate biopsy;
- patients without multiparametric prostate MRI;
- patients with a PI-RADS score 1 or 2;
- technical errors from the image fusion;
- patients who were not given prophylactic antibiotics or enema;
- unwillingness or inability to sign an informed consent form.

### 2.2. MRI Acquisition and Categorization for Registration

Multiparametric MRI was performed before the biopsy in all patients. Antiperistaltic drugs (Buscopan; Boehringer Ingelheim, Ingelheim am Rhein, Germany) were administrated intramuscularly 30 min before MRI examination. MRI was performed using a 3.0 T machine (Ingenia CX; Philips, Amsterdam, The Netherlands) with a phased array cardiac 6-channel coil. The protocol included three orthogonal planes of T2-weighted imaging (T2WI), diffusion-weighted imaging (DWI), and dynamic contrast-enhanced MRI (DCE-MRI). The detailed parameters of the T2WI were as follows: TR, 2500–3000 ms; TE, 70–90 ms; slice thickness, 3 mm; interslice gap, 0 mm; field of view, 160 mm × 160 mm; matrix, 320 × 320; and number of excitations, 1. Diffusion encoding gradients with *b*-values of 0, 100, 1000, 1500 s/mm^2^ were applied. Apparent diffusion coefficient maps were automatically generated on a pixel-by-pixel basis.

An experienced uroradiologist, blinded from all clinical information, categorized the level of suspicion for clinically significant cancer using PI-RADS (version 2.1) from 1 to 5, as follows: grade 1, highly unlikely to be present; grade 2, unlikely to be present; grade 3, equivocal; grade 4, likely to be present; and grade 5, highly likely to be present. A single index lesion with score over 3 was chosen for TB in each patient. After locating the lesion with DWI and DCE-MRI, axial T2WI was used for image registration for its superior spatial resolution, regardless of the location of the index lesion.

### 2.3. Image Fusion, Registration, and Error Measurements 

All image fusions were performed by another experienced uroradiologist who did not participate in lesion categorization. We used a RS85 Prestige (Samsung Medison, Seoul, Korea) US system fitted with an end-firing transrectal probe (EA2-11AR). The fusion imaging technique (S-Fusion) uses an electromagnetic tracking system consisting of an electromagnetic field transmitter near the patient and an electromagnetic sensor attached to a US transducer. A fully integrated position sensor unit installed in the US machine receives the position and orientation data from the sensors. Before the examinations in our study, axial T2WIs were uploaded from the picture archiving and communication system archive to the US machine.

**Figure 1:**
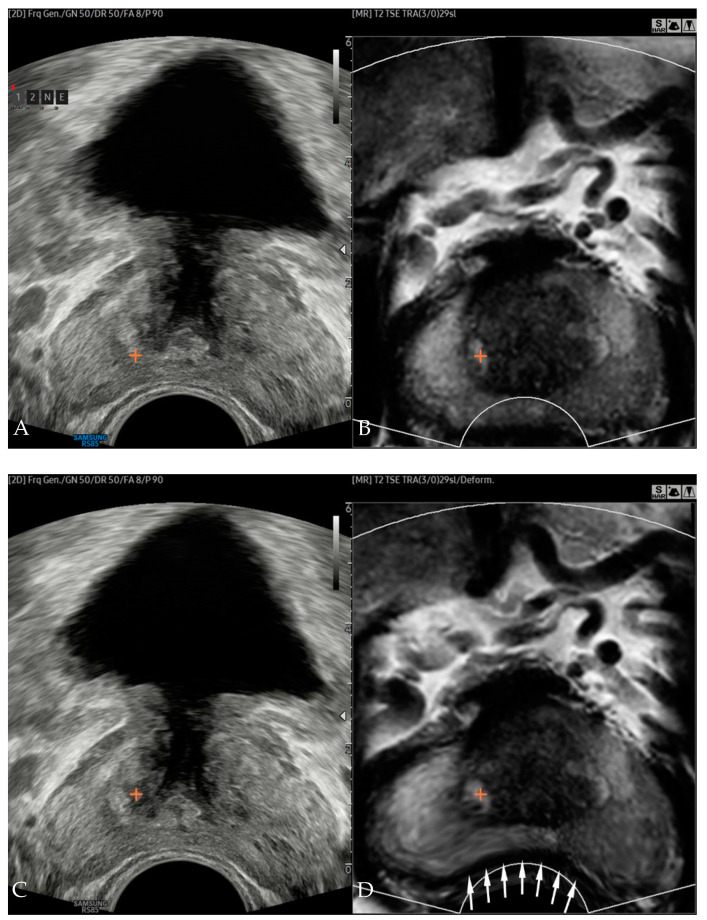
Side-by-side display of US (**A**) and MRI (**B**) of a 65-year-old male after rigid registration. Cross-mark drawn on MRI transferred to the corresponding point on US. Similar to the US (**C**) with a transrectal probe inserted, MRI (**D**) demonstrated concavely deformed posterior margin (arrows) after real-time corrected nonrigid registration. Internal image features of MRI were also deformed. Cross-mark selected on the deformed MRI also moved to its new corresponding point in the US.

After uploading the MRI, the prostate contour was automatically segmented slide by slide, and prostate volume was calculated. Rigid registration by plane was performed using the outer contour of the prostate, the location of the seminal vesicle, the shape of the transition zone, the shape of urethra, and the apex as landmarks. Once the US image showed similar imaging features with those of the MRI, we finished the rigid registration. After the image registration, a reference lesion with similar location was chosen by the examined radiologists on MRI, and the marking was transferred to the US (Figure 1A,B).

The 2D points in the US image coordinates were converted into 3D points in MRI coordinates. Then, the trans-registration error (TRE) was measured from the Euclidian distance between marked lesions on US and MRI in 3D MRI space. Real-time nonrigid registration was performed immediately after the TRE measurement. Image deformation was completed in less than one second, and new marks indicating reference point appeared on the deformed MRI and US (Figure 1C,D). TRE was re-measured for nonrigid registered images. A more detailed description of the technique was published in a previous study [10].

### 2.4. Prostate Biopsy

**Figure 2:**
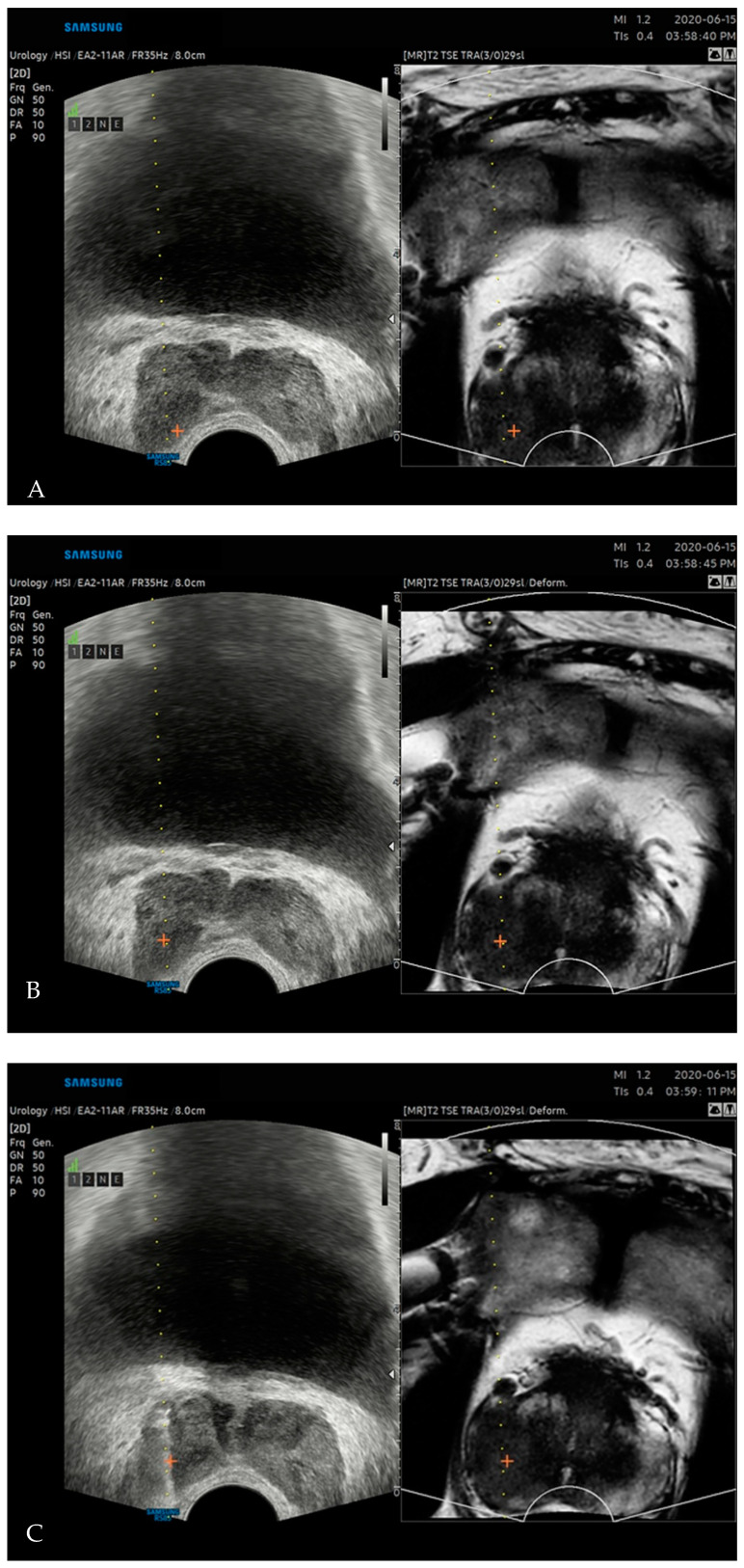
Fusion prostate biopsy of a 64-year-old male. Rigid registration between US and MRI (**A**) was performed, and index lesion was marked as cross on MRI, and transferred to the US. After nonrigid registration, deformed MRI and repositioning of index lesion markers in US were noted (**B**). Adenocarcinoma with a Gleason score of 8 (4 + 4) was confirmed by targeted biopsy performed on the index lesion (**C**). Trans-registration error decreased from 4.78 to 0.46 mm by switching from rigid to nonrigid registration.

Transrectal US-guided biopsy was performed by the same uroradiologist who had conducted the image fusion. Local anesthesia with 10 mL of lidocaine was applied before each biopsy. An automated biopsy gun with an 18-gauge, 20 cm cutting needle (ACECUT, TSK Laboratory, Tochigi, Japan) was used. Initially, rigid registration of US and MRI were performed (Figure 2A). After registration, the cross-mark on the index lesion on the MRI was transferred to the corresponding US point. Then, nonrigid image registration was performed (Figure 2B). Slight change of markers position was noted after nonrigid registration. Under the guidance of the nonrigid registered US and MRI, two-core TB per each index lesion was performed (Figure 2C), followed by 12-core SB. For each biopsy core, Gleason score and length of PCa, if any, were recorded by an experienced uropathologist blinded from the clinical results.

### 2.5. Outcome Measures

The primary outcome was registration accuracy. TRE from rigid and nonrigid registrations were measured and compared. The secondary outcome included clinically significant cancer detection rates (CDR) and prostate-volume measurement. CDR was assessed on a per patient and per core basis, and analyzed according to PI-RADS score. Clinically significant cancer was defined as any core of Gleason score of 7 or higher, or two or more cores with a Gleason score 6 confirmed by biopsy. During the image registration process, prostate volume was automatically measured using MRI, and compared with manual US measurement using standard ellipsoid formula (width × height × length × pi/6).

### 2.6. Sample Number Calculation and Statistical Analysis

We expected the mean TRE from rigid and nonrigid registration to be 8 and 5 mm, respectively, based on our in-house phantom study. With 80% statistical power and a two-sided significance level of 5%, we calculated the number of patients to be enrolled as 16. Assuming a conservative dropout rate of 20% for sample size calculation, a final sample size of 20 patients was calculated. Comparisons of mean TREs and volumes were performed with the Wilcoxon signed-rank test. A Bland–Altman plot was drawn to reveal a relationship between the differences and the magnitude of volume measurements. The CDRs of TB and SB were compared with a chi-squared test. Sample size was calculated using PASS 15.0.6 software (NCSS, LLC. Kaysville, UT, USA) and other statistical analyses were performed using MedCalc 19.5.6 software (MedCalc, Mariakerke, Ostend, Belgium).

## 3. Results

### 3.1. Patient Characteristics

A total of 19 patients were enrolled with a median age of 67 years (range: 57–83 years). The mean ± standard deviation (SD) of prostate-specific antigen level was 9.7 ± 7.3 ng/mL (range: 2.0–34.7 ng/mL).

Descriptive patients’ characteristics are summarized in Table 1.

### 3.2. Comparison of Transregistration Error from Rigid and Nonrigid Registration

**Figure 3:**
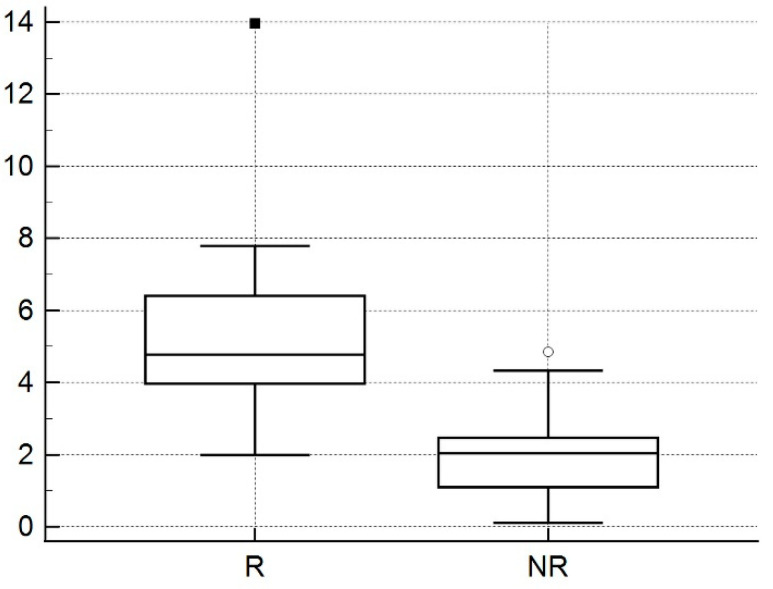
Box and whisker plot of mean trans-registration error (TRE). TRE from nonrigid registration (NR) was statistically smaller than TRE from rigid registration (R).

Mean ± SD of TREs were 5.32 ± 2.61 mm (range: 1.99–13.95 mm) and 2.11 ± 1.37 mm (range: 0.11–4.84 mm) for rigid and nonrigid registrations between MRI and US, respectively (*p* < 0.05) (Figure 3). There was no statistical difference of TRE to the locations and anatomical zones of index lesion.

### 3.3. Per Patient and Per Core Cancer-Detection Rates

Clinically significant PCa was detected in 51.6% of the patients (10/19). Nine out of ten patients with clinically significant PCa (90.0%) showed positive results from both TB and SB. In the one remaining patient, only SB detected clinically significant PCa, while TB failed.

Eight (42.1%) patients had an index lesion of PI-RADS 3, five (26.3%) patients had an index lesion of PI-RADS 4, and six (31.6%) patients had an index lesion of PI-RADS score 5. Table 2 summarizes the CDRs by patient according to PI-RADS score.

The CDR by core was 25.2% (67/266). For TB, cancer was found in 44.7% (17/38) of biopsy cores, which was significantly higher than 21.9% (50/228) for SB (*p* < 0.05).

### 3.4. Comparison of Prostate Volume from MRI and Transrectal US

**Figure 4:**
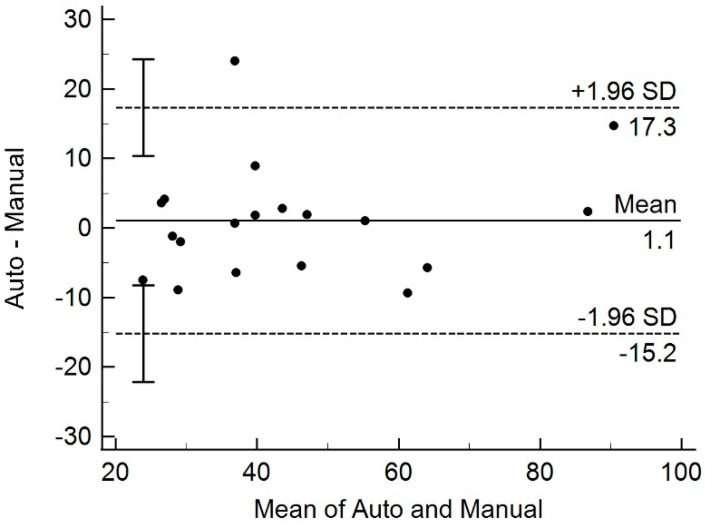
Bland–Altman plot of prostate volume measured by automatic and manual methods. All but one measurement differed within 1.96 standard deviation, indicating no systematic variation between the two measurements.

Mean ± SD prostate volume was 45.2 ± 20.5 mL (range: 20.2–97.8 mL) as automatically measured on MRI, and 44.1 ± 19.0 mL (range: 24.6–85.5 mL) as measured manually in the US (*p* = 0.89) (Figure 4).

## 4. Discussion

The introduction and popularization of multiparametric prostate MRI is changing the biopsy paradigm, as US-guided biopsy often fails to detect aggressive PCa [11,12]. Previous studies showed that the probability of detecting clinically significant PCa increases with higher scores when prostate MRI is analyzed using the Likert scale or PI-RADS categorization [13,14]. Therefore, direct MRI-guided biopsy or cognitive or software-assisted MRI-US fusion biopsy can be more helpful than the conventional US-guided biopsy in determining prostatectomy [15,16]. Fusion biopsy is a more convenient option, but since errors occurring in the process of registering two different types of images are inevitable, efforts are being made to reduce them.

Trans-registration error is a measure of Euclidian distance between cross-marked anatomically homologous points on US and MRI, such as urethra, calcification and, in this case, PCa. It measures the distance between the landmark on MRI and the corresponding point mapped to the MRI from US, in 3D MRI space [10]. Our results showed that TRE decreased with the transition from rigid to nonrigid registration. A mean TRE of 2.11 mm implies that this technique can precisely register lesions that are smaller than 5 mm in size. The TRE in this prospective study was smaller than that (2.98 mm) obtained from patient data, and closer to that (1.60 mm) calculated from the phantom in the previous retrospective study [10].

For real-time error compensation in fusion biopsy, a few studies used a two-stage registration strategy using an initial preoperative 3D US to 3D MRI registration, followed by intraprocedural 2D US to 3D US registration [12,16]. However, for these techniques, a 3D probe or 3D US reconstruction from 2D US sweep is essential. A transrectal 3D probe costs more, causes patient discomfort due to its large caliber, and takes much longer for the reconstruction of preoperative registration data. A 3D US reconstruction from a 2D US sweep does not need additional instruments, but reliable and constant sweeping is hard to achieve and varies by operators. Overall TREs from these studies were higher than ours, require longer reconstruction time to reconstruct, and are phantom-based [17].

Clinical validations of elastic fusion using 3D transrectal probe were published [9]. Moldovan et al. reported that their registration error was suitable for clinical practice. Co-registration accuracy was significantly influenced by the operator’s experience, and was poorer in the anteroposterior direction and at the apex. However, these results were evaluated retrospectively, from the virtual biopsy using fiducial marker insertion to the patients. Cornud et al. compared cognitive and elastic fusion using 3D US. They concluded that, as the cognitive method is based on robust anatomic landmarks, it is prone to inaccuracy because the MRI is orthogonal, whereas transrectal US is oblique in terms of image acquisition plane. The elastic fusion registration, developed to circumvent this limitation, showed a striking advantage in precision compared with the cognitive technique.

However, an up-to-date meta-analysis demonstrated that rigid and nonrigid image registrations showed similar CDRs for clinically significant or all PCa. They indirectly compared rigid and nonrigid registered TBs by comparing each method with SB, and showed no significant difference in CDRs between the two types of TB and SB (*p* = 0.83) [18]. Another clinical comparison study with rigid versus nonrigid registration was performed by Sokolakis [8]. They consecutively assessed two platforms with rigid image registration (BioJet, D&K Technologies and UroNav, Invivo Corporation, Paris, France) and one with nonrigid registration (Trinity, KOELIS, Princeton, NJ, USA). They also failed to demonstrate significant differences among the three fusion biopsy systems in terms of the highest International Society of Urological Pathology Grade Group (*p* > 0.99). One important message from their conclusion was that the rigid registration is easier to use and needs shorter operation time compared to nonrigid method (16–17 versus 28 min). This means that currently commercially available elastic fusion could be more accurate, but clinical results are not sufficient to show the differences, so the operator cannot afford the increase in procedural time and accompanying inconvenience.

Our real-time nonrigid fusion technique has several advantages. First, a dedicated 3D US transducer is not required. Since our fusion technique is deep-learning-software-based, no additional hardware is required other than the electromagnetic field generator system currently used for rigid registration. Second, real-time nonrigid fusion is operated with just one click, in less than a second. Although we did not measure the generation time for nonrigid registration in this study, our previous reports showed that mean run time was only 112 ms [10]. In addition to the superb increase in accuracy, this convenience can be readily welcomed by the clinicians.

Our study showed clinically significant CDR of 52.6% by patient. This finding is heavily attributed to the demographics of our patients. Eight out of nineteen (42.1%) patients had PI-RADS 3 lesions, for which clinically significant PCa was equivocally present by definition. No cancer was detected in this group. On the other hand, cancer was detected in 10 out of 11 (90.9%) patients with PI-RADS 4 and 5 lesions. In the one remaining patient, the cancer not found in TB was diagnosed through SB, from the same sector as the targeted lesion, suggesting a targeting failure. Therefore, SB should not be omitted when performing TB. This is in line with a study by Rapisarda et al. who found that agreement on final histological reporting was higher when SB was additionally performed on TB [19]. 

Measuring prostate volume in patients with suspicious for prostate cancer is very important. For the risk stratification of cancer, PSA density or PSA divided by prostate volume is a reliable biomarker. In a recent article, Massanova et al. [20] reported that the actual surgical specimen weight correlated well with prostate volume on MRI, either by automatic segmentation software or by manual measurement, but not with transrectal US measurement. In our study, automatically measured prostate volume after loading the axial MRIs shows good agreement with manually measured volume from US. Only one patient showed a difference in volume measurement beyond two standard deviations. Considering the inaccuracies from transrectal US volume measurement by ellipsoid formula, the automatic measurement from MRI would be more accurate, although it needs to be further verified by comparing it with the volume of surgical specimen.

The limitations of this study are as follows. First, although the cases were prospectively collected according to a precalculated sample number, this calculation is just about TRE estimations between two registration techniques and not for the secondary outcome including CDR. This study, however, is based on patients who were consecutively referred for biopsy over a certain period of time, and patients with PI-RADS score 3 or more were selected. Therefore, CDR should be appreciated with caution, and not be generalized. Second, since the study population was relatively small, further randomized studies with larger populations would be required to demonstrate CDR from nonrigid registration is superior to that from rigid registration. Third, pathological confirmation was based on biopsy cores, not whole mounts of sections from radical prostatectomy specimens that can correspond with MRI. With this reference standard, we cannot tell whether targeting accuracy is sufficient in some cases, because even a lesion with PI-RADS score 5 may not necessarily be cancerous. Fourth, our solution does not address large out-of-plane errors, which require feature-to-feature- based registration.

## 5. Conclusions

Our real-time nonrigid registration technique decreases registration errors. Deformation of MRI was very quick and easy, and can increase accuracy of lesion targeting. Further studies with large populations comparing the CDRs of nonrigid versus rigid registration are required.

## Figures and Tables

**Table 1:** Descriptive patients’ characteristics in the study.

	Mean	Standard Deviation
Age (years)	67.7	8.0
PSA (ng/mL)	9.7	7.3
Prostate colume by TRUS (mL)	44.1	19.0
Prostate volume by automatic measurement (mL)	45.2	20.5
	**Frequency**	**Percent**
Preoperative Gleason score (*n* = 11)		
3 + 3	2	18.2
3 + 4	2	18.2
4 + 3	5	45.5
4 + 4	2	18.2
Postoperative Gleason score (*n* = 7)		
3 + 4	1	14.3
4 + 3	5	71.4
4 + 5	1	14.3
pT stage (*n* = 7)		
2c	2	28.6
3a	4	57.1
3b	1	14.3

Abbreviations: PSA: prostate-specific antigen; TRUS: transrectal ultrasound.

**Table 2:** Cancer detection rates by patients according to PI-RADS score.

PI-RADS Score	CSC Patient	SB Positive	TB Positive
3 (*n* = 8)	0 (0%)	0 (0%)	0 (0%)
4 (*n* = 5)	4 (80.0%)	4 (80.0%)	4 (80.0%)
5 (*n* = 6)	6 (100%)	6 (100%)	5 (83.3%)

Abbreviations: PI-RADS: prostate imaging-reporting and data system; CSC: clinically significant cancer; SB: systemic biopsy; TB: targeted biopsy.

## Data Availability

The data are not publicly available for the viewpoint of protecting personal information.
